# Neurons in the Pigeon Nidopallium Caudolaterale Display Value-Related Activity

**DOI:** 10.1038/s41598-018-23694-8

**Published:** 2018-03-29

**Authors:** Madeline Dykes, Aylin Klarer, Blake Porter, Jonas Rose, Michael Colombo

**Affiliations:** 1 Department of Psychology, University of Otago, Dunedin, New Zealand; 2 Fakultät für Psychologie, Ruhr-Universität, Bochum, Germany

## Abstract

We recorded from neurons in the nidopallium caudolaterale, the avian equivalent of the mammalian prefrontal cortex, in four birds. The birds were required to peck a stimulus that indicated the amount of reward they would receive (small or large) after a certain delay (short or long). We found that the activity of neurons in the nidopallium caudolaterale was modulated by the value of the reward that would be received based on the reward amount and the delay to reward. We found that value coding was most prominent during the presentation of the sample period, and less so during the delay period and during the presentation of the reward itself. Our findings support the view that activity in nidopallium caudolaterale reflects the encoding of the value of reward based on a combination of reward amount and delay to a reward.

## Introduction

Temporal discounting is the diminishing perception of the effects of an action due to a delay^1^. Frontal areas of the human brain, and in particular the orbitofrontal cortex (OFC), have been implicated in temporal discounting^2,3^. Some cells in OFC, for example, fire in relation to reward magnitude, some encode the length of the delay, while others only fire in increasing anticipation of a reward delivery^4^. Cells in the OFC of primates are also known to modulate their activity in response to changes in the value (benefits minus costs) of a reward, achieved by varying the duration of the delay^5,6^.

In the current study we investigated whether cells in the avian brain also code the value signified by a stimulus, and whether that value is modulated by the delay to receive the reward. In line with studies in mammals indicating that value coding occurs in the frontal areas of the brain, we examined the activity of cells in the nidopallium caudolaterale (NCL), the avian analogue of the primate prefrontal cortex^7,8^. Recently, a number of studies have begun to examine the reward processing characteristics of NCL cells. Koenen, Millar, and Colombo^9^ found that NCL cells were modulated by whether a stimulus predicted a large, small, or no reward. Specifically, in the period where the cue was presented, and in the subsequent delay period before the delivery of the reward, some NCL cells exhibited a graded change in activation as a function of the anticipated reward amount.

Cells in the NCL also seem to be involved in temporal discounting. Kalenscher *et al*.^10^ gave pigeons a choice between an immediate small reward and an immediate large reward. Naturally, the pigeons chose the immediate large reward, but across the session, as the delay to the large reward was increased, pigeons shifted their preference to the immediate small reward. The NCL cells integrated reward amount and delay-to-reward, thereby coding the subjective reward value of a stimulus^10^.

The findings of Koenen *et al*.^9^ and Kalenscher *et al*.^10^ suggest that cells in NCL code stimulus value, and that the value may be modulated by the delay to reward. These experiments, however, have two limitations. First, in both studies, pigeons were trained using a choice paradigm, and as such, many factors could be at play in modulating neural activity. When two stimuli appear on a screen, for example, any modulation in firing could be in response to either of the stimuli. Second, the neural activity was examined during the delay period, and again, factors other than pure stimulus value may have come into play in modulating neural activity. Neural modulation in the delay period, for example, could be a result of the visual components of the stimulus eliciting continued responses that carry over into the delay period that are unrelated to the value that the subject assigns to the stimulus. Similarly, neural modulation during the delay period could also result from movements made by the animals in anticipation of receiving a reward, again potentially contaminating any neural modulation based on the value the animal assigns to the stimulus.

Perhaps a better way to examine value coding is to observe neural activity while an animal is presented with a single stimulus that indicates the magnitude of the reward to be received following a specified delay period. Such an experiment has recently been conducted by Kasties, Starosta, Güntürkün, and Stüttgen^11^, who measured neural activity in NCL when pigeons were presented with one of four stimuli that predicted one of four conditions: small reward after a short delay, small reward after a long delay, large reward after a short delay, or large reward after a long delay. Although Kasties *et al*.^11^ did find cells in the NCL that responded differently to the four stimuli, a fact perhaps not too surprising given the known visual sensitivity of NCL cells^12^, they found no evidence that the responses to the stimuli were modulated by either the reward amount, the delay length, or the interaction of reward amount and delay length. The authors concluded that the cells in NCL showed no evidence of value coding^11^.

Given that NCL cells code reward amount^9^, temporal discounting^10^, and the shift in preference from a delayed large reward to a small immediate reward^10^, it is surprising that Kasties *et al*.^11^ found no evidence that NCL cells were modulated by reward value. There are, however, two limitations of the Kasties *et al*.^11^ study that may have made it difficult to find value coding. The first limitation concerns aspects of the reward itself. In the Kasties *et al*.^11^ study, the small reward consisted of 1–1.5 s access to food and the large reward consisted of 5–6 s access to food. The variable nature of the reward duration with the “small” and “large” categories, plus the fact that reward was only delivered on 50% of the trials, may have interfered with the mapping of a reward value onto an associated stimulus.

A second limitation of the Kasties *et al*.^11^ study was that value coding was only assessed during the presentation of the stimulus. A number of investigators have found that information concerning the value of an item is transmitted during the period between stimulus presentation and the appearance of the reward. For example, Komura *et al*.^13^ found that when presented with a cue signalling a preferred reward, cell firing in the rat’s thalamus increased across the delay period subsequent to stimulus presentation. Similarly, Koenen *et al*.^9^ reported differential firing in NCL cells in response to differing reward amounts during a delay period prior to reward. By focusing on just the sample period, Kasties *et al*.^11^ may have underestimated the capacity of NCL cells to engage in value coding.

Another important issue that Kasties *et al*.^11^ did not investigate was whether value coding continued into the reward period. In mammals, the OFC has been implicated in updating information related to expected outcome important for decision making, thereby facilitating flexible behaviour^6,14^. Past experiments have found that OFC activity is modulated by reward value at the time of reward presentation^4,15^. To date, while NCL activity during the reward period has been recorded^16^, whether that activity is modulated by the perceived value of the stimulus that preceded it has not been explored.

In the current study we re-examined the issue of value coding in NCL neurons. We trained pigeons to associate four cues with four different conditions; short delay followed by small reward (S1), short delay followed by large reward (S3), long delay followed by small reward (L1) and a long delay followed by large reward (L3). Instead of the stimuli predicting reward on 50% of the trials, the stimuli in the current experiment predicted reward on 100% of the trials. Additionally, Epstein’s (1981)^17^ finding that the amount of food gained is not necessarily proportional to the duration of the food presentation, and that long food access durations run the risk of depleting the food hopper^18^, has prompted some to manipulate reward amount by using a specified number of reward-presentation periods, rather than different reward durations^19–21^. In line with these findings, our small reward consisted of one 2-s period of food delivery and our large reward consisted of three 2-s periods of food delivery. Finally, we examined value coding in NCL activity in the sample period, the delay period, and the beginning of the reward period.

## Methods

### Subjects

The subjects were four experimentally naïve adult homing pigeons (*Columba livia*). The birds had free access to grit and water and were fed a mixture of wheat, corn, peas, pellets, and grains sufficient to maintain them at 80–85% of their free feeding body weight. They were housed in individual wire-mesh cages and kept on a 12 to 12 light-dark cycle with lights on at 07:00 h. The subjects were kept and treated in accordance with the University of Otago Code of Ethical Conduct for the Manipulation of Animals, and the University of Otago Animal Ethics Committee approved the experiment.

### Apparatus

The subjects were trained and tested in standard operant chambers measuring 35 cm (length), 43 cm (width), and 39 cm (height). At the front of the chamber was a 43 cm monitor surrounded by an infrared touch frame. In front of the touch frame sat a plexiglas panel with six square holes arranged in a 2-row x 3-column format. The size of each square was 6 cm by 6 cm and the center-to-center distance of each hole was 6.5 cm. Situated 20 cm below the center key was a hopper that could be illuminated and delivered the wheat reward. The stimuli consisted of four black and white pictures; one picture of Arnold Schwarzenegger, one picture of a cactus flower, one picture of a black crow and one picture of a person on a skateboard at a skate park. The stimuli were 6 cm by 6 cm in size, and appeared centered in the square aperture.

### Behavioural Task

**Figure 1:**
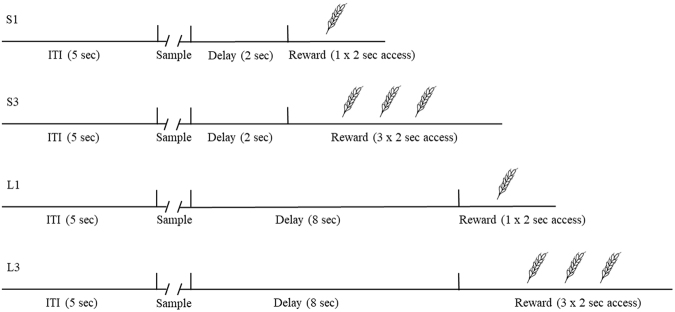
Behavioural procedure. The sequence of events for each of the four stimuli. S1: short delay, small reward; S3: short delay, large reward; L1: long delay, small reward; and L3: long delay, large reward. The small reward consisted of one 2-s period of wheat reward, whereas the large reward consisted of three 2-s periods of wheat reward. The short delay and long delays were 2 s and 8 s in duration, respectively.

At the end of a 5-s intertrial interval (ITI), one of the four stimuli appeared on the center-top hole (Fig. 1). Three pecks to the stimulus turned it off and initiated a delay period. Every peck to the touchscreen was accompanied by a 50-ms 1000-Hz feedback tone. At the end of the delay period the reward was delivered. Each stimulus was followed by either a short (2 s) delay or a long (8 s) delay, and either a small reward (one 2-s period of access to wheat) or large reward (three 2-s periods of access to wheat). For the large reward the three 2-s periods were delivered one after the other with an inter-reward pause of 1 s during which time the hopper was lowered. For ease of exposition, the stimuli are referred to as S1, S3, L1 and L3, with the letters signifying a short or long delay, and the number signifying one or three reward periods. Each session consisted of 64 trials, with 16 trials dedicated to each of the four stimuli, randomly intermixed. Which stimuli were associated with each of the four delay/reward conditions was balanced across animals. The birds were trained until the latency to peck the S3 stimulus was shorter than the latency to peck the L1 stimulus across four of five consecutive sessions.

### Surgery

At the completion of behavioral training the birds were implanted with a lightweight microdrive to allow single-unit recording. They were first anesthetized with a mixture of ketamine (25 mg/kg) and xylazine (5 mg/kg). After pruning the feathers on the scalp and overlaying the ears, the head was immobilized using a Revzin stereotaxic adapter^22^. The scalp was first sprayed with a topical anesthetic (10% xylocaine) and then cut and retracted to expose the skull. A hole was drilled above the NCL at AP +5.5 ML ±7.5^23^. Six stainless steel screws were placed into the skull (one serving as a ground screw), the tips of the electrodes of the microdrive were lowered to position them above the NCL, and the microdrive was secured to the skull with dental acrylic. The incision was sutured and sprayed with xylocaine. The bird recovered in a padded and heated cage until fully alert and mobile and then returned to its home cage where it was allowed to recover for at least seven days before the recording sessions started.

### Neural Recording

Eight 25-um formvar-coated nichrome wires (California Fine Wire, Grover Beach, CA, USA) mounted in the microdrive were used for recording the extracellular activity of single neurons in the NCL. For each session one electrode was used to record the neural activity and a second electrode with minimal activity served as the indifferent. All electrodes were impedance–matched to about 0.5–1 MOhm. The signal was passed through a FET headstage, then a Grass P511K preamplifier (Grass Instruments, Quincy, Massachusetts, United States) where it was amplified and filtered to remove mains electrical 50 Hz noise. An oscilloscope and speaker were used to monitor the signals. A CED micro1401 system (Cambridge Electronic Design Limited, Cambridge, United Kingdom) collected the electrophysiological data, and CED Spike2 software was used for behavioral time-tagging of all events and analysis of the spike data. Good isolation of a cell, and a signal-to-noise ratio of at least 2:1, were the criteria for selecting a cell for a recording session. After cell isolation the behavioural program was initiated. A recording session lasted approximately one hour. At the end of the session the electrodes were advanced 40 µm. The birds were tested once a day.

### Data Analysis

We analyzed the sample period (Sample), the first second of the delay (Delay-1), the second second of the delay (Delay-2), and the first second of the reward period (Reward-1) for evidence of value coding in the firing rates of NCL neurons. With respect to the sample period, unlike studies with primates where one can monitor the exact position of the eyes, it is difficult in pigeons to monitor when they are looking at the visual stimulus. Colombo, Frost, and Steedman^24^ adopted a convention that neural activity to a viewed stimulus was measured during a period from −400 ms to −100 ms prior to the first peck to that stimulus. The reason the period ends 100 ms prior to contact with the keys is because pigeons close their eyes approximately 80 ms prior to a key peck^25^.

Cells were selected for population plot analysis in one of two ways. In the first selection method each cell’s Sample, Delay-1, and Delay-2 data was subjected to a one-way repeated-measures ANOVA with Stimulus (4: S1, S3, L1, L3) as a factor. The dependent variable was the firing rates of cell during the 300 ms sample presentation periods or 1000 ms delay periods. In the second selection method we conducted a paired t-test comparing the firing rates of the cells during the S3 and L1 trials, again separately for the sample and the two delay periods. On the basis of whether the main effect of Stimulus was significant the cell’s data entered into a population plot.

For the population plots, each cell’s data was normalized by dividing all its firing rates by the average firing in the middle three seconds of the ITI period, and subjected to two-way repeated-measures ANOVAs with Stimulus (4: S1, S3, L1, L3) and Bin (6: bins 1–6 for the sample period, or 20: bins 1–20 for the delay period) as factors, with repeated measures over Bins (Greenhouse-Geisser corrected). LSD planned pairwise comparisons were evaluated at *p* < 0.05.

## Results

### Histology

**Figure 2:**
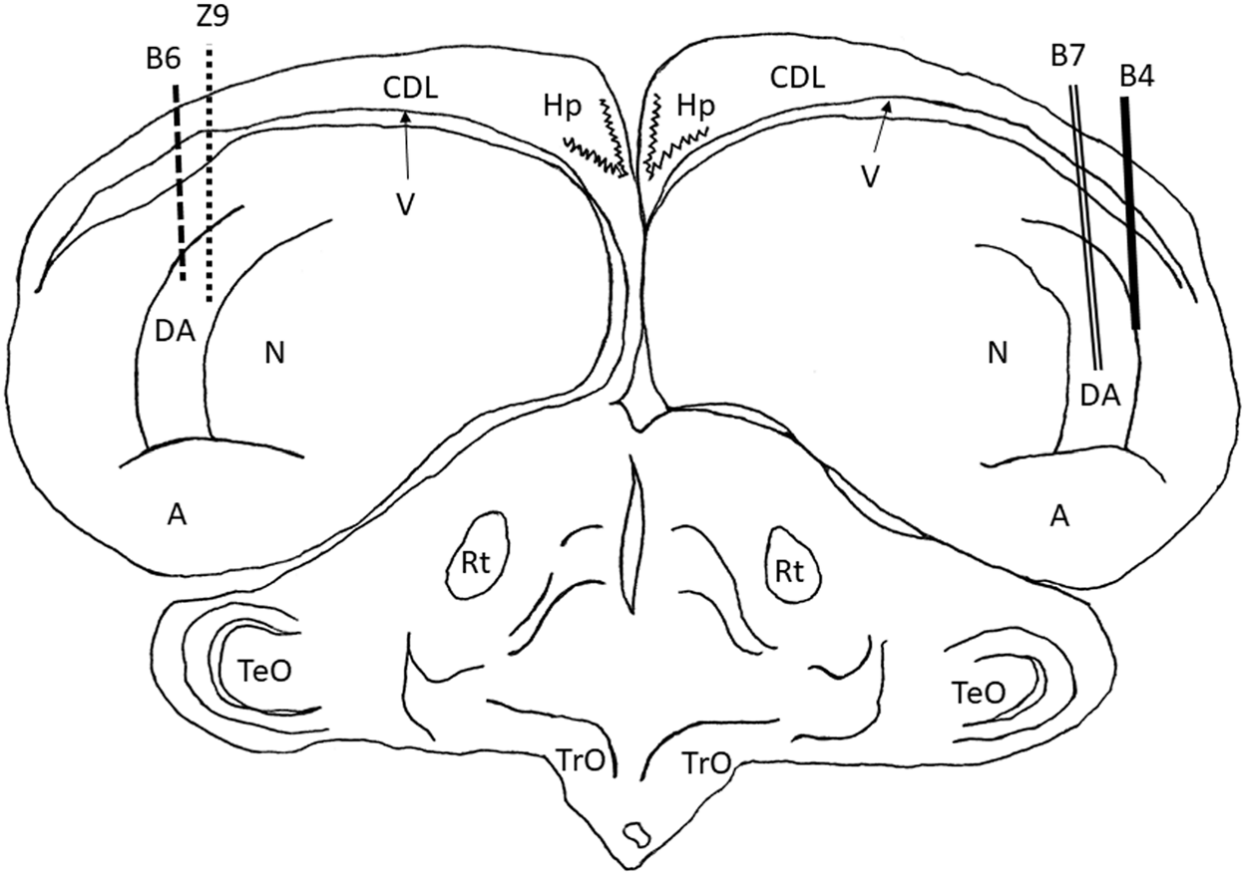
Electrode track reconstruction. Electrode track position reconstructions for the two right NCL birds (B4 and B7) and two left NCL birds (B6 and Z9). All recordings were within the full dorsal-ventral extent of NCL. The following are the brain regions as defined by Reiner *et al*.^26^: A, archopallium; DA, tractus dorso-arcopallialis; CDL, area corticoidea dorsolateralis; Hp, hippocampus; N, nidopallium; Rt, nucleus rotundus; TeO, tectum opticum; TrO, tractus opticus; V, ventricle.

All electrode tracks were within the targeted NCL region as defined by Karten and Hodos^22^. The histology results are shown in Fig. 2. The intended track positions for NCL were AP +5.5 and ML +7.5. The track position for the first left NCL bird (Z9) was AP +6.75 and ML +7.5, differing only from the intended AP position by 1.25 mm. The track position for the second left NCL bird (B6) was AP +5.5 and ML +7.8, differing only from the intended ML position by 0.3 mm. The track position for the first right NCL bird (B4) was AP +6.0 and ML +7.6, differing from the intended AP position by 0.5 mm and the intended ML position by 0.1 mm. The track position for the second right NCL bird (B7) was AP +5.75 and ML +7.2, differing from the intended AP position by 0.25 mm and the intended ML position by 0.3 mm.

### Behavioural Data

**Figure 3:**
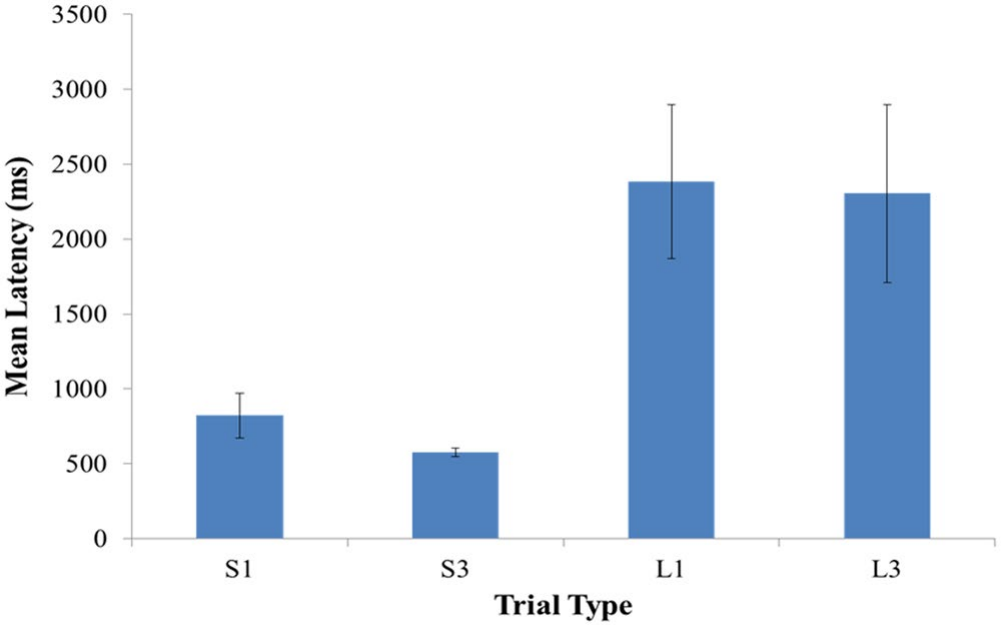
Behavioural performance. Mean latency of the first peck to each of the four stimuli for the four birds averaged across all sessions from which neurons were isolated. Note that shorter latencies represent higher value to the pigeon. Error bars represent ± 1 SEM.

For each session, the median latency to the first peck was calculated across the 16 trials dedicated to each of the four stimuli. The latency to the first peck averaged across all sessions from which cells were recorded is displayed in Fig. 3. The latencies were subjected a log_10_ transformation to reduce across-subject variability, and the transformed latencies were subjected to a one-way repeated-measures ANOVA with Stimulus (4: S1, S3, L1, L3) as the factor (Greenhouse-Geisser corrected). There was a significant effect of Stimulus, *F*(3, 9) = 43.49, *p* < 0.01. Planned pairwise comparison confirmed the latencies to S1 and S3 differed from the latencies to L1 and L3, but that the latencies to S1 and S3 were no different from each other, nor did the latencies for L1 and L3 differ from each other. The failure to detect a difference between S1 and S3 was due to one of the four subjects who, although showed faster responses to the stimuli that predicted the short delay (S1 and S3) than the long delay (L1 and L3), showed no difference in its latency to S1 and S3.

### Sample Period

**Figure 4:**
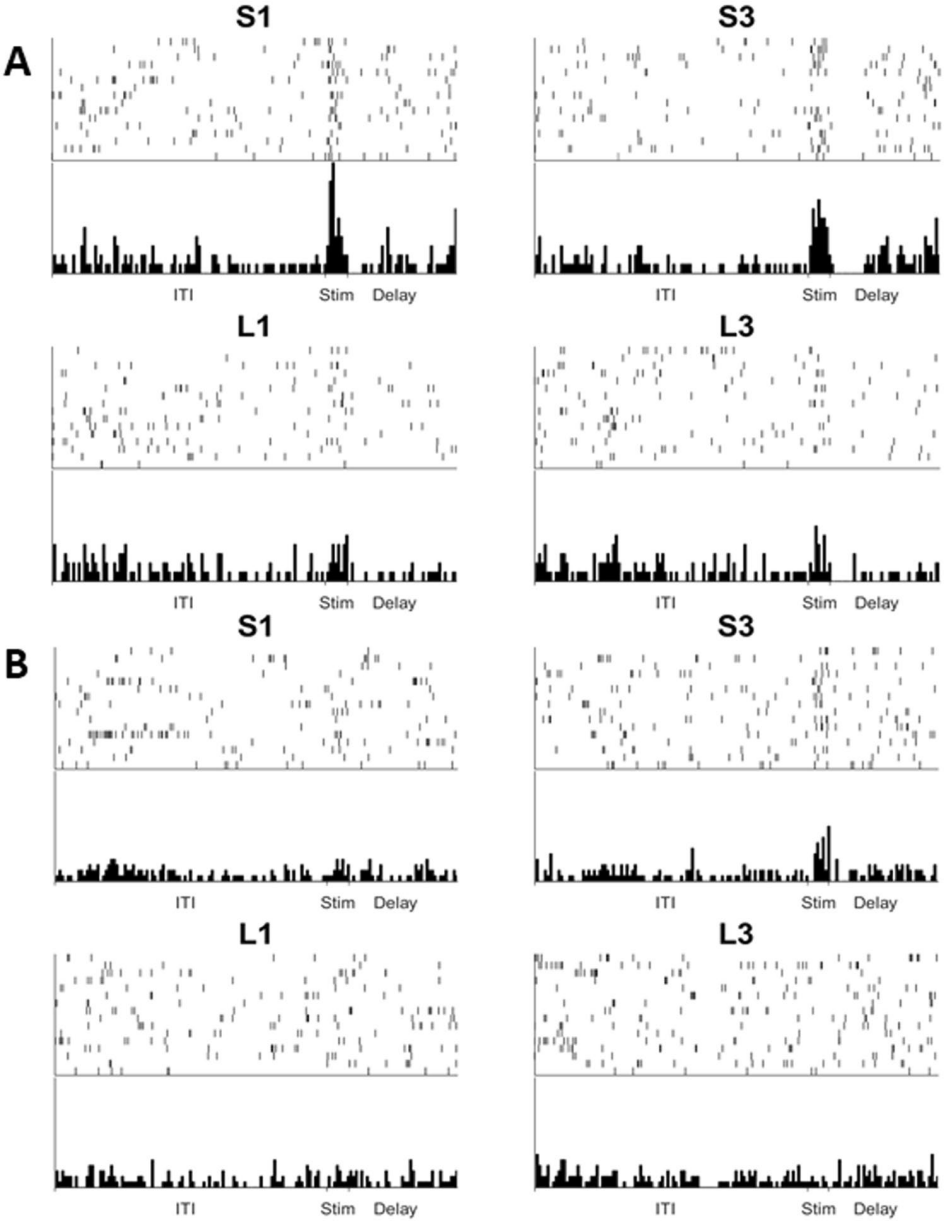
Examples of NCL cell activity. Panel A and B show two different cells, each from different birds. Both panels present the raster and histogram of one cell over 64 trials. Panel A shows a cell that exhibits an increased firing rate at the time that the stimulus is presented for all four stimuli, however there is a noticeably increased response to S3 and S1 compared to L3 and L1. Panel B shows a cell that only exhibits an increase in firing on the presentation of the S3 stimulus, with no change in firing seen when the other stimuli were presented.

We recorded from a total of 207 cells. There were no right-left differences in the response characteristics of the cells and so the data were collapsed across this variable. Of the 207 cells, 35 displayed a significant effect of Stimulus (one-way ANOVA) during the 300 ms Sample period. Examples of two cells that responded differently to the stimuli during the sample period are shown in Fig. 4.

**Figure 5:**
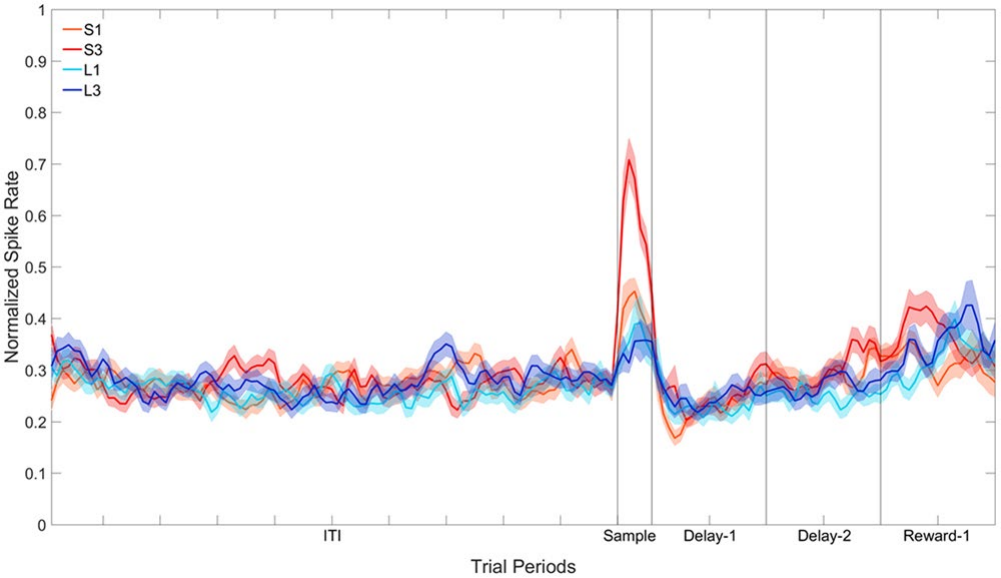
Population plot: Sample filtering (peck aligned). Normalised firing rate for cells that show a significant effect of Stimulus across the four trial types during the Sample period. The ITI represents the entire 5 s ITI period, Sample represents a 300 ms period from −400 ms to −100 ms prior to the first peck, and Delay-1 and Delay-2 represent the first second and second second of the 2 s (for S1 and S3) or 8 s (for L1 and L3) delay period. Reward-1 is the first second of reward presentation. In S1 and S3, therefore, Reward-1 occurs after the 2 s delay period, whereas in L1 and L3 Reward-1 occurs after the 8 s delay period.

The population response for all 35 cells that showed a significant effect of Stimulus is shown in Fig. 5. The data in Sample, Delay-1, Delay-2, and Reward-1 periods were subjected to separate two-way repeated-measures ANOVA with Stimulus (4: S1, S3, L1, L3) and Bin (6: bins 1–6 for the Sample period, or 20: bins 1–20 for the delay and reward periods) as factors, with repeated measures over Bins (Greenhouse-Geisser corrected). In the Sample period there was a significant effect of Stimulus, *F*(3, 102) = 4.98, *p* < 0.01. Planned pairwise comparisons revealed that the activity to S3 was greater than the activity to all the other stimuli, the activity to S1 was greater than the activity to L1 and L3, however there was no difference in the activity between L1 and L3. In the Delay-1 and Delay-2 periods, there was no significant effect of Stimulus, all *F*s(3, 102) < 1.76, all *p*s > 0.17.

Overall, in the Reward-1 period there was also no significant effect of Stimulus, *F*(3, 102) = 1.90, *p* = 0.16. We analysed the first 500 ms of the reward period as well and did find a significant effect of Stimulus, *F*(3, 102) = 3.53, *p* < 0.05. Pairwise comparisons revealed that activity to S3 was greater than the activity to L1, but not S1 or L3. There were no differences in activity between S1, L1 and L3. In the second 500 ms of the reward period, there was no significant effect of Stimulus, *F*(3, 102) = 1.99, *p* = 0.15.

**Figure 6:**
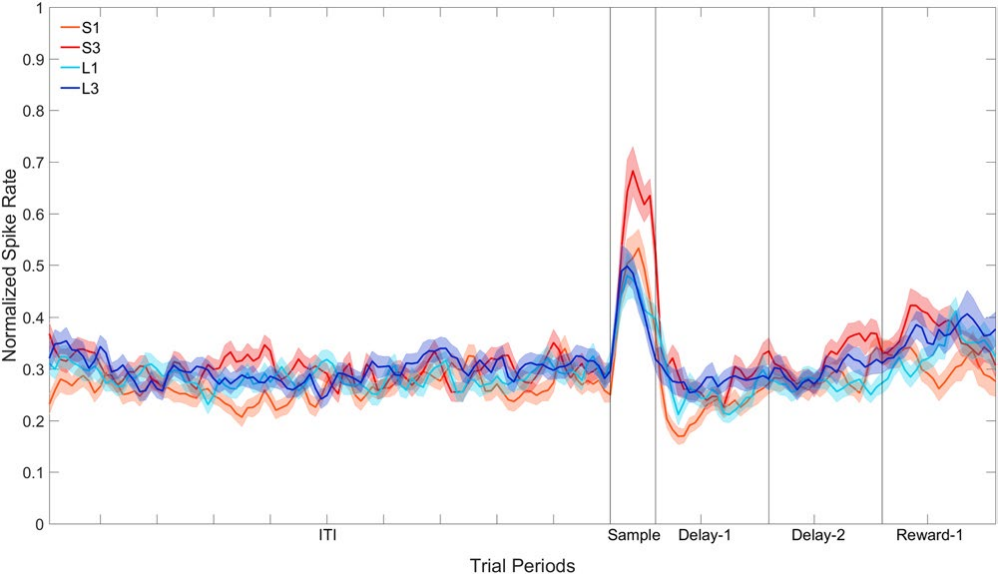
Population plot: Sample filtering (stimulus onset aligned). Normalised firing rate for cells that show a significant effect of Stimulus across the four trial types during the Sample period. The ITI represents the entire 5 s ITI period, Sample represents a 400 ms period immediately following the onset of the stimulus, and Delay-1 and Delay-2 represent the first second and second second of the 2 s (for S1 and S3) or 8 s (for L1 and L3) delay period. Reward-1 is the first second of reward presentation. In S1 and S3, therefore, Reward-1 occurs after the 2 s delay period, whereas in L1 and L3 Reward-1 occurs after the 8 s delay period.

Recall that the Sample period analysed corresponded to a 300 ms period from −400 ms to −100 ms prior to the first peck (see Methods). Although restricting the analysis to this period has certain advantages, such as it is uncontaminated by pecks and, more importantly, we are sure that the bird is looking at the stimulus during this period, it does have one disadvantage. Given the latencies to the stimuli that predicted short delays (S1 and S3) were just over 0.5 s, and the latencies to the stimuli that predicted the long delays (L1 and L3) were just over 2 s, the “sample” period was, relative to their onset, much earlier for the S1 and S3 stimuli compared to the L1 and L3 stimuli. We therefore conducted a second analysis on the 35 cells examining value coding in a 400 ms period from the onset of the stimuli, excluding trials for which the latency to respond to the stimulus was less than 400 ms. The results are shown in Fig. 6.

The data in the Sample period was subjected to two-way repeated-measures ANOVA with Stimulus (4: S1, S3, L1, L3) and Bin (6: bins 1–6) as factors, with repeated measures over Bins (Greenhouse-Geisser corrected). In the Sample period there was a significant effect of Stimulus, *F*(3, 102) = 4.43, *p* < 0.05. Identical to the previous analysis based on a sample period from −400 ms to −100 ms prior to the first peck, when the sample period consisted of the first 400 ms from the onset of the stimuli, planned pairwise comparisons revealed that the activity to S3 was still greater than the activity to all the other stimuli, and that the activity to L1 and L3 were no different to each other. In contrast to the previous analysis, the activity to S1 was no different to the activity to L1 and L3. In summary, the lower response rate to the L1 and L3 stimuli observed in the previous analysis was not due to the fact that the “sample” period for these stimuli was much later than for the S3 stimulus.

We also examined the overall preferences for each of the 35 cells individually (see Data Analysis). Of the 35 cells, 30 responded to S3 and at least one of the other three stimuli (S1, L1, and L3). Of these 30 cells, 24 (80%) showed a clear and significant (*p* < 0.05) preference for S3 over the remaining stimuli. Thus not only is the preference for S3 exhibited at a population-level analysis, but it is also present in a tally of the individual cells’ preferences.

### Delay-1 Period

**Figure 7:**
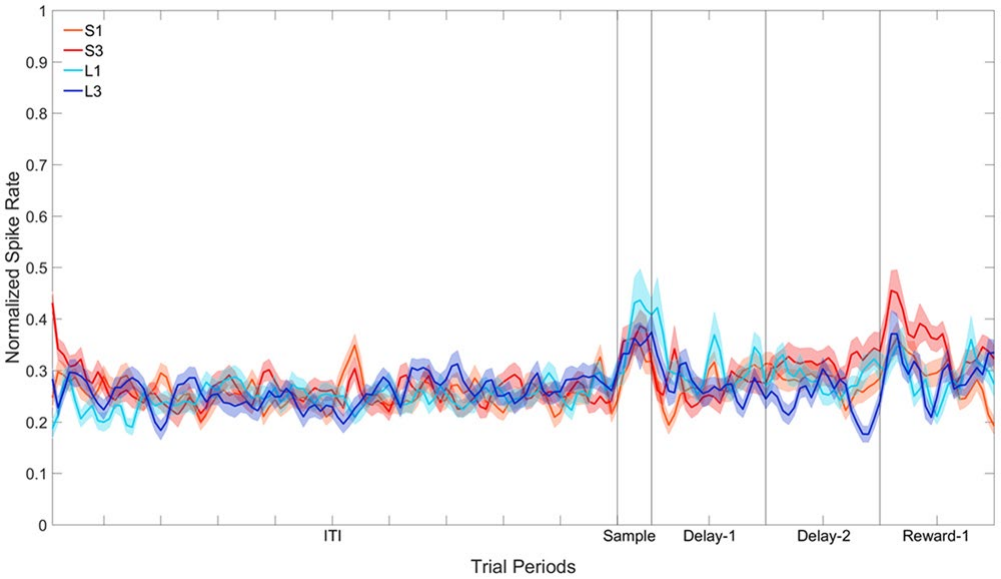
Population plot: Delay-1 filtering. Normalised firing rate for cells that show a significant effect of Stimulus across the four trial types during the Delay-1 period. The ITI represents the entire 5 s ITI period, Sample represents a 300 ms period, and Delay-1 and Delay-2 represent the first and second second of the 2 s (for S1 and S3) or 8 s (for L1 and L3) delay period. Reward-1 is the first second of reward presentation. In S1 and S3, therefore, Reward-1 occurs after the 2 s delay period, whereas in L1 and L3 Reward-1 occurs after the 8 s delay period.

Of the 207 cells, 25 displayed a significant effect of Stimulus (one-way ANOVA) during the Delay-1 period. Note that these 25 cells were not selected on the basis of whether they show enhanced activity during the delay, but simply on the basis of whether they showed *differential* activity to the four stimuli during the delay. Of these 25 cells, nine overlapped with those that showed a significant effect of Stimulus in the Sample period. The population response for these 25 cells is shown in Fig. 7. The data in the Sample, Delay-1, Delay-2 and the first second of the reward period were subjected to separate two-way repeated-measures ANOVA with Stimulus (4: S1, S3, L1, L3) and Bin (6: bins 1–6 for the Sample period, or 20: bins 1–20 for the delay and reward periods) as factors, with repeated measures over Bins (Greenhouse-Geisser corrected). In neither the Sample period, Delay-1 period, or Delay-2 period, was there was a significant effect of Stimulus, all *F*s(3, 72) < 1.49, all *p*s > 0.26.

Overall, in the Reward-1 period there was a significant effect of Stimulus, *F*(3, 72) = 3.82, *p* < 0.05. Pairwise comparisons revealed that activity to S3 differed from activity to all other stimuli. Activity in S1, L1 and L3 did not differ. We also analysed the first 500 ms of the reward period and again noted a significant effect of Stimulus, *F*(3, 72) = 4.75, *p* < 0.01. Pairwise comparisons revealed that activity to S3 differed from activity to both L1 and L3, but not S1. There was no difference between activity to S1 and any other stimulus, and the activity to L1 and L3 did not differ from one another. In the second 500 ms of the reward period, there was no significant effect of Stimulus, *F*(3, 72) = 1.75, *p* = 0.17.

### Delay-2 Period

**Figure 8:**
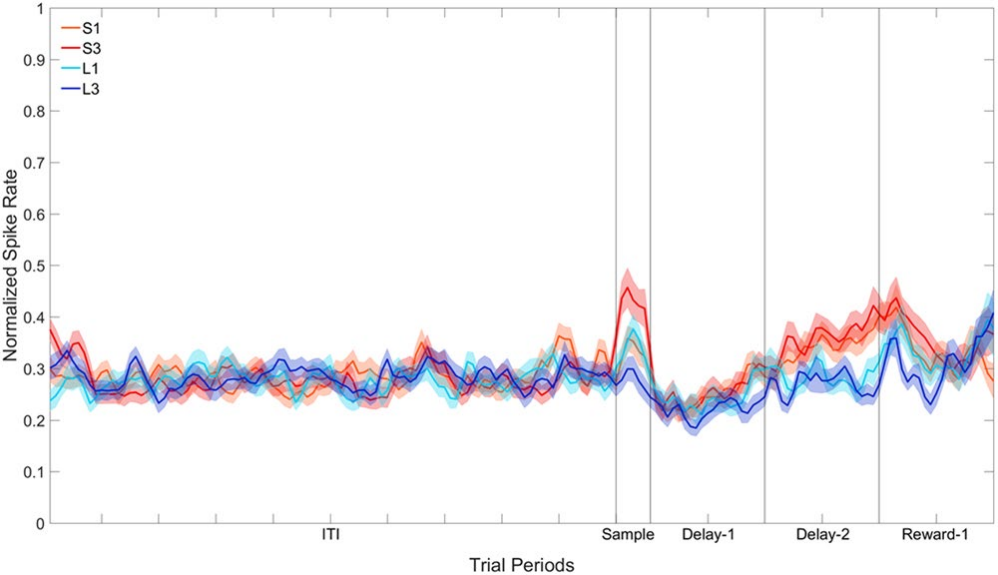
Population plot: Delay-2 filtering. Normalised firing rate for cells that show a significant effect of Stimulus across the four trial types during the Delay-2 period. The ITI represents the entire 5 s ITI period, Sample represents a 300 ms period, and Delay-1 and Delay-2 represent the first and second second of the 2 s (for S1 and S3) or 8 s (for L1 and L3) delay period. Reward-1 is the first second of reward presentation. In S1 and S3, therefore, Reward-1 occurs after the 2 s delay period, whereas in L1 and L3 Reward-1 occurs after the 8 s delay period.

Of the 207 cells, 39 displayed a significant effect of Stimulus (one-way ANOVA) during the Delay-2 period. Note that as was the case in the Delay-1 analysis, these 39 cells were not selected on the basis of whether they show enhanced activity during the delay but simply on the basis of whether they showed *differential* activity to the four stimuli during the delay. Of these 39 cells, four overlapped with those that showed a significant effect of Stimulus in the Sample period, three overlapped with those that showed a significant effect in the Delay-1 period, and five overlapped with those that showed a significant effect of Stimulus in both periods. The population response for these 39 cells is shown in Fig. 8. The data in the Sample, Delay-1 and Delay-2 period were subjected to separate two-way repeated-measures ANOVA with Stimulus (4: S1, S3, L1, L3) and Bin (6: bins 1–6 for the Sample period, or 20: bins 1–20 for the delay and reward periods) as factors, with repeated measures over Bins (Greenhouse-Geisser corrected). In the Sample period, there was a significant effect of Stimulus, *F*(3, 111) = 4.57, *p* < 0.05. Planned pairwise comparisons (*p* < 0.05) revealed that S3 differed from S1 and L3, but not L1. S1 differed from S3 and L3, but not L1, and L1 did not differ from any of the stimuli. There was no significant effect of Stimulus in the Delay-1 and Delay-2 periods, all *F*s(3, 111) < 2.28, all *p*s > 0.10.

Although we failed to find any evidence of value coding in the Delay-2 period, an inspection of Fig. 8 reveals the beginnings of differential coding towards the end of the Delay-2 period. To examine the effect further, we examined the last 500 ms of the Delay-2 period. The data was subjected to a two-way repeated-measures ANOVA with Stimulus (4: S1, S3, L1, L3) and Bin (10: bins 1–10 for the delay period) as factors, with repeated measures over Bins (Greenhouse-Geisser corrected). There was a significant effect of Stimulus, *F*(3, 114) = 3.44, *p* < 0.05. Planned pairwise comparisons (*p* < 0.05) revealed that firing to S1 differed from firing to L1 and L3, while there was no difference in firing between S1 and S3. Firing to S3 differed significantly from L3, and the difference between S3 and L1 neared significance. L1 and L3 did not differ from one another.

Overall, in the Reward-1 period there was no significant effect of Stimulus, *F*(3, 114) = 0.07, *p* = 0.36. We also analysed the first 500 ms of the reward period and again there was a significant effect of Stimulus, *F*(3, 114) = 3.37, *p* < 0.05. Planned pairwise comparisons (*p* < 0.05) revealed that there was no difference in firing among S3, S1 and L1 and the activity to L3 differed from the activity to all other stimuli. In the second 500 ms of the reward period, there was no effect significant of Stimulus, *F*(3, 114) = 0.22, *p* = 0.85.

## Discussion

### Summary of Findings

In the present study, we explored how cells in NCL encode value as an integrative sum of reward amount and delay to reward. We recorded from 207 NCL cells during a task where birds were required to peck a stimulus that predicted either a small or large reward, following either a short or long delay period. We examined the firing of these cells during the period that the birds were presented with each stimulus, and in the delay period prior to reward.

When cells where filtered on the basis of showing a significant Stimulus effect in the Sample period, 35 of 207 cells (16.9%) fired in a pattern that closely mirrors the birds’ stimulus preferences (note that a shorter response latency to a stimulus indicated that the birds valued the stimulus higher). Specifically, the birds responded fastest to the stimulus that predicted a short delay followed by a large reward (S3) over the stimulus that predicted a short delay followed by a small reward (S1), and both of these stimuli were preferred over the two stimuli that predicted a long delay (L3 and L1) which were no different from each other. The neural data in the Sample period of these 35 cells mirrored these stimulus preferences. When the period immediately following the stimulus presentation was analysed, cells still fired in preference for S3, however the differentiation between S1 and the other stimuli disappeared. Given that it cannot be guaranteed that the bird is looking at the stimuli at this time, the first analysis used is likely to be a more accurate representation of the cell’s response to the stimulus. Despite evidence for value coding in the Sample period, the stimulus preferences of these sample-selective cells were not carried over into either the Delay-1 or Delay-2 periods. However, in the first 500 ms of the reward period, these cells fired significantly more to S3 than to L1. The difference in firing was not evident in the second 500 ms of the reward period.

When cells were filtered based on activity in the Delay-1 period, 25 of 207 cells (12.0%) showed a significant effect of Stimulus. There was no difference in firing to any of the four stimuli in the Sample, Delay-1, or Delay-2 periods of these 25 cells. In other words, although a particular cell might have shown a preference for one stimulus over another, a different cell would have shown a different pattern of preference, such that when all these cells were combined, as in our population plot, no clear stimulus preference emerged. The firing of cells filtered based on the Delay-1 period did however show some difference in firing in the first 500 ms of the reward period. Cells fired more to S3 than to either L1 or L3, but the difference in firing disappeared in the second 500 ms of the reward period.

Finally, when the cells were filtered based on activity in the Delay-2 period, 39 of 207 cells (18.8%) displayed a significant effect of Stimulus. Interestingly, these 39 cells that were selected on the basis of their firing during the Delay-2 period did display some evidence of value coding in the Sample period in that the cells fired significantly more to S3 than to all stimuli except L1. Similar to the situation for the Delay-1 period, there was no difference in firing to any of the four stimuli in the Delay-1 or Delay-2 periods. However, we found that differentiation did occur to some extent in the last 500 ms of the Delay-2 period in that the neural responses to S1 and S3 were no different to each other, S1 differed from both L1 and L3, but S3 only differed from L3 and the difference between S3 and L1 neared significance. In other words, cell firing in the last half of the delay-2 period began to differentiate between short and long delay. While there was no evidence of actual value coding from the Delay-2 period cells, the change in firing pattern seems to reflect anticipation of reward.

There was also very little evidence for value coding in the reward period. The only evidence for value coding was in the first 500 ms of the reward period expressed generally as an increased firing to S3, but the effect was weak. By the second 500 ms of the reward period there was no evidence of any value coding.

### Comparison to Other Studies

Previous studies showed that NCL appears to encode reward amount^9^ as well as subjective reward value of a stimulus by integrating reward amount and delay-to-reward in choice paradigms^10^. In both of these studies, the NCL activity showed modulation during the delay period prior to the delivery of the reward. The findings of the current study support the role of NCL in detecting value of conditioned stimuli and rate coding the “best” option (short delay-large reward) during the Sample period when the bird is shown the stimulus. Our study also finds that the modulation of NCL activity based on reward amount and delay-to-reward is not exclusive to choice paradigms. Instead, NCL cells react in response to a single stimulus that indicates a reward and its temporal cost, so any change in activity cannot be explained by factors that could be at play in choice paradigm, such as an integrated value assessment based on being presented with two different options.

Previously, NCL activity has been found to be modulated by reward delivery^9,16^. At the most, the firing patterns observed in the current study in the reward period reflected to some extent the “best” reward outcome when cells were filtered by activity in the Sample and the Delay-1 periods. The fact that activity reflected the best option is consistent with findings of neural activity of mammalian OFC. The OFC is thought to play an important role in updating information about expected rewards^4,15^. Our finding that activity of NCL cells during reward delivery was modulated to some extent by the value of a reward suggests that NCL may play a role similar to the OFC in updating expected reward outcomes to optimise future behaviours.

Our findings also have implications for a recent study by Kasties *et al*.^11^, whose design was on the surface similar to that of the current study, yet failed to find any evidence for value coding in NCL. They found that while NCL cells responded differently to four different stimuli, there was no evidence of modulation in NCL activity in response to reward amount, delay length, nor an interaction of reward and delay length. The absence of value coding in NCL neural activity occurred despite the fact that, based on latencies, their subjects showed clear preferences for the stimuli that were similar to that of our own birds. In contrast, we did find modulation in NCL activity in response to stimuli that predicted different reward amounts. What may have accounted for the differences between the two studies? We incorporated a number of small changes that we believe assisted the pigeons to differentiate between the different stimuli and the reward outcomes they predicted. First, in contrast to Kasties *et al*.^11^ who delivered reward on only 50% of trials, we delivered reward on 100% of trials. Furthermore, in contrast to Kasties *et al*.^11^ who manipulated reward amount by increasing the duration for which access to food was available, we manipulated reward amount by increasing the number of reward presentation periods. We believe that by increasing the reward delivery periods, and by delivering reward on 100% of the trials, the birds were better able to determine the “best” option. As a result, we saw that in both the behavioural data, and in the neural data during the Sample period, NCL cells clearly reflected the most valuable outcome, followed by the next most valuable outcome.

Our finding of no value coding in the delay seems to stand in contrast to that of Koenen *et al*.^9^, who reported reward modulation during the delay period. Closer inspection of our data, however, revealed the emergence of reward modulation in the last half of the Delay-2 period. The fact that Koenen *et al*.^9^ reported more evidence of reward modulation in the delay period may be due to the fact that they employed a 3 s delay whereas we only analyzed a 2 s delay period. More to the point, it is more likely that the activity we began to witness towards the end of the Delay-2 period, and that observed by Koenen *et al*.^9^ is better classified as activity related to the anticipation of reward than value coding. The reason in the case of the current study is that the increase in neural activity was observed on short delay trials irrespective of whether there were one (S1) or three (S3) rewards imminent. Thus, the neural activity in the last half of the Delay-2 period was more likely coding an upcoming reward rather than the value of the recently-seen stimulus. The same outcome was reported by Koenen *et al*.^9^ in the no-choice condition, where reward modulation was observed in that the cells displayed much less firing to 0 upcoming rewards than to 1 or 3 upcoming reward, the latter two being neurally indistinguishable. It thus appears that reward modulation in the delay is more a representation of whether a reward is imminent, than a code for the value of the recently seen stimulus.

### Implications for Value Coding in NCL

The literature supporting NCL as a functional analogue to the mammalian PFC, at least with respect to value coding, is relatively small. Based on the mammalian literature the PFC, and in particular the OFC, is involved in encoding value. Studies of the mammalian OFC find that firing is modulated by reward magnitude and in anticipation of reward delivery^2–4^. Furthermore, OFC activity is modulated in response to changes in reward value achieved by manipulating the delay to reward only at the presentation of a stimulus, during the delay period and when reward is delivered^5,6^. To date, the research in the avian brain has confirmed that NCL is implicated in encoding reward amount and in encoding delay to reward^9,10^. The present study adds to the current knowledge in that NCL appears to show properties similar to the OFC with respect to encoding value based on both delay to reward and reward amount. We can now add value coding relating to temporal discounting to the list of functional similarities with the mammalian PFC.

## References

[1] Critchfield TS, Kollins SH (2001). Temporal discounting: basic research and the analysis of socially important behavior. J. App. Behav. Anal..

[2] Bechara A (2003). Risky Business: Emotion, decision-making, and addiction. J. Gambl. Stud..

[3] Horn N, Dolan M, Elliott R, Deakin J, Woodruff P (2003). Response inhibition and impulsivity: an fMRI study. Neuropsychologia.

[4] Roesch MR, Taylor AR, Schoenbaum G (2006). Encoding of time-discounted rewards in orbitofrontal cortex Is independent of value representation. Neuron.

[5] Roesch MR, Olson CR (2005). Neuronal activity in primate orbitofrontal cortex reflects the value of time. J. Neurophysiol..

[6] Padoa-Schioppa C, Assad JA (2006). Neurons in the orbitofrontal cortex encode economic value. Nature.

[7] Güntürkün O (2005). Avian and mammalian “prefrontal cortices”: Limited degrees of freedom in the evolution of the neural mechanisms of goal-state maintenance. Brain Res. Bull..

[8] Güntürkün O (2005). The avian ‘prefrontal cortex’ and cognition. Current Opinion in Neurobiology.

[9] Koenen C, Millar J, Colombo M (2013). How bad do you want it? Reward modulation in the avian nidopallium caudolaterale. Behav. Neurosci..

[10] Kalenscher T (2005). Single units in the pigeon brain integrate reward amount and time-to-reward in an impulsive choice task. Curr. Biol..

[11] Kasties N, Starosta S, Güntürkün O, Stüttgen MC (2016). Neurons in the pigeon caudolateral nidopallium differentiate Pavlovian conditioned stimuli but not their associated reward value in a sign-tracking paradigm. Sci. Rep.UK.

[12] Scarf D, Stuart M, Johnston M, Colombo M (2016). Visual response properties of neurons in four areas of the avian pallium. J. Comp. Physiol. A.

[13] Komura Y (2001). Retrospective and prospective coding for predicted reward in the sensory thalamus. Nature.

[14] Schoenbaum G, Roesch M (2005). Orbitofrontal cortex, associative learning, and expectancies. Neuron.

[15] Rolls ET, Kringelbach ML, Araujo IETD (2003). Different representations of pleasant and unpleasant odours in the human brain. Eur. J. Neurosci..

[16] Kalt T, Diekamp B, Güntürkün O (1999). Single unit activity during a go/nogo task in the “prefrontal cortex” of pigeons. Brain Res..

[17] Epstein R (1981). Amount consumed as a function of magazine-cycle duration. Behav. Anal. Letters.

[18] Epstein R (1985). Amount consumed varies as a function of feeder design. Journal of the Experimental Analysis of Behavior.

[19] Davison M, Baum WM (2000). Choice in a variable environment: every reinforcer counts. J. Exp. Anal. Behav..

[20] Elliffe D, Davison M, Landon J (2008). Relative reinforcer rates and magnitudes do not control concurrent choice independently. J. Exp. Anal. Behav..

[21] Landon J, Davison M, Elliffe D (2003). Concurrent schedules: reinforcer magnitude effects. J. Exp. Anal. Behav..

[22] Karten, H. W. & Hodos, W. *A Stereotaxic Atlas of the Brain of the Pigeon (Columba Livia)*. Baltimore: Johns Hopkins University Press. (1967)

[23] Kröner S, Güntürkün O (1999). Afferent and efferent connections of the caudolateral neostriatum in the pigeon (*Columba livia*): A retro- and anterograde pathway tracing study. J. Comp. Neurol..

[24] Colombo M, Frost N, Steedman W (2001). Responses of ectostriatal neurons during delayed matching-to-sample behavior in pigeons (*Columba livia*). Brain Res..

[25] Goodale MA (1983). Visually guided pecking in the pigeon *(Columba livia)*. Brain Behav. Evolut..

[26] Reiner A (2004). Revised nomenclature for avian telencephalon and some related brainstem nuclei. J. Comp.Neurol..

